# Association of Midlife Inflammatory Markers With Cognitive Performance at 10-Year Follow-up

**DOI:** 10.1212/WNL.0000000000201116

**Published:** 2022-11-15

**Authors:** Teemu Kipinoinen, Sini Toppala, Juha O. Rinne, Matti H. Viitanen, Antti M. Jula, Laura L. Ekblad

**Affiliations:** From the Turku PET Centre (T.K., S.T., J.O.R., L.L.E.), University of Turku and Turku University Hospital, Finland; Kuopio City Home Care (S.T.), Rehabilitation and Medical Services for Elderly, Kuopio, Finland; City of Turku (M.H.V.), Welfare Division, Department of Geriatrics, Turku City Hospital and University of Turku, Finland; Division of Clinical Geriatrics (M.H.V.), NVS, Karolinska Institutet, Stockholm, Sweden; and National Institute for Health and Welfare (A.M.J.), Turku, Finland.

## Abstract

**Background and Objectives:**

Chronic low-grade inflammation, commonly associated with cardiovascular diseases and risk factors, has been associated inconclusively with cognitive decline and dementia. The aim of our study was to evaluate whether low-grade inflammation, measured in midlife, is associated with a decline in cognitive performance after a 10-year follow-up. We hypothesized that low-grade inflammation, estimated by interleukin-6 (IL-6), tumor necrosis factor alpha (TNF-α), and high-sensitivity CRP (hs-CRP), is a predictor of cognitive decline in the general population.

**Methods:**

This prospective cohort study is based on a Finnish nationwide, population-based Health 2000 Examination Survey, its supplemental examinations in 2000–2001, and the follow-up Health 2011 Survey. Cognitive performance at baseline and at follow-up was assessed with categorical verbal fluency (VF), word-list learning (WLL), and word-list delayed recall (WLDR). Baseline low-grade inflammation was measured with IL-6, TNF-α, and hs-CRP in 2001. Associations between low-grade inflammation and cognitive performance were analyzed with multivariable linear models adjusted for age, sex, education, *APOE ε4* genotype, type 2 diabetes, hypertension, hypercholesterolemia, body mass index, depressive symptoms, smoking, and baseline cognition.

**Results:**

Nine hundred fifteen participants aged 45–74 years (median age 54 years, 55% women) were included in the analysis. Both higher IL-6 and TNF-α at baseline predicted poorer performance in VF and WLL at 10-year follow-up (VF: IL-6 β: −1.14, *p* = 0.003, TNF-α β: −1.78, *p* = 0.008; WLL: IL-6 β: −0.61, *p* = 0.007, TNF-α β: −0.86, *p* = 0.03). Elevated IL-6 also predicted a greater decline in VF and WLL after a 10-year follow-up (VF: β: −0.81, *p* = 0.01; WLL: β: −0.53, *p* = 0.008). Baseline TNF-α did not predict cognitive decline, and hs-CRP did not predict cognitive performance or decline after 10-years.

**Discussion:**

Our results suggest that low-grade inflammation in midlife is an independent risk factor for poorer cognitive performance later in life. Of the studied markers, IL-6 and TNF-α seem to be stronger predictors for cognitive performance and decline than hs-CRP.

Low-grade inflammation is associated with a number of disorders including cardiovascular disease, obesity, metabolic syndrome, depression, and dementia.^1-6^ However, it is unclear whether low-grade inflammation is the cause or consequence of these conditions.^3,7^ There is also evidence that levels of circulating inflammatory markers are elevated before the onset of cognitive impairment or Alzheimer disease (AD),^8-10^ although some studies have not been able to find association between inflammatory markers and cognitive decline.^11,12^ However, few prospective studies have assessed the roles of different inflammatory markers during midlife as risk factors for cognitive impairment later in life.^9,10,13,14^ Furthermore, many of the previous studies have been cross-sectional^15-17^ or have investigated older populations instead of middle-aged populations^2,8,11,12,18-23^ resulting in heterogeneity in outcomes. To date, the importance of low-grade inflammation in cognitive decline and dementia remains inconclusive.

It has been suggested that chronic inflammation is promoted by cytokines such as TNF-α and IL-6 excreted from adipose tissue.^24-29^ The presence of these circulating cytokines is hypothesized to activate microglia cell–mediated neuroinflammation in the brain that is believed to be part of the pathophysiology of AD.^30^ We have previously shown that higher levels of metabolic risk factors and high-sensitivity C-reactive protein (hs-CRP) are associated with microglial activation, measured with [^11^C]PBR28 -PET, in brain regions typical for early β-amyloid accumulation in AD.^31^ The hypothesis of the present study was that low-grade inflammation in midlife would be associated with cognitive performance and decline later in life. To test this hypothesis, we examined 915 individuals aged 45–74 years at baseline and evaluated whether higher levels of 3 circulating proinflammatory mediators, TNF-α, IL-6, and hs-CRP, could be associated with cognitive decline after 10 years. The study was conducted on a prospective cohort design in a sample of the Finnish adult population.

## Methods

### Study Population

This study is based on the Finnish Health 2000 examination survey, its supplemental examinations on a subpopulation in 2001–2002, and its follow-up survey, Health 2011 study, all of which were conducted by the Finnish Institute for Health and Welfare.^32,33^ A multidisciplinary epidemiologic Health 2000 survey was conducted in Finland in the years 2000 and 2001. It was a nationwide population-based survey that was representative of the Finnish adult population living in mainland Finland. A total of 8028 adults, aged 30 years and older, were randomly selected from the Finnish population registry using a 2-staged stratified cluster sampling procedure. Eighty-four percent (n = 6,770) attended a health examination proper or had a health examination at home.^33^

After the Health 2000 examinations, a subsample of the study population underwent further examinations between 2001 and 2002 for a more thorough study of cardiovascular diseases and diabetes. Of the participants aged 45–74 years who had participated in the Health 2000 examination and lived near 1 of the 5 central university hospital cities in Finland, 1864 were invited to a supplemental investigation of the cardiovascular system. Eighty-two percent (n = 1,526) attended the supplemental survey.^34^ The participants in the original Health 2000 Survey, who were still living in Finland and who had not declined to participate (n = 1,396), were invited to attend the Health 2011 follow-up survey.^33^

**Figure 1:**
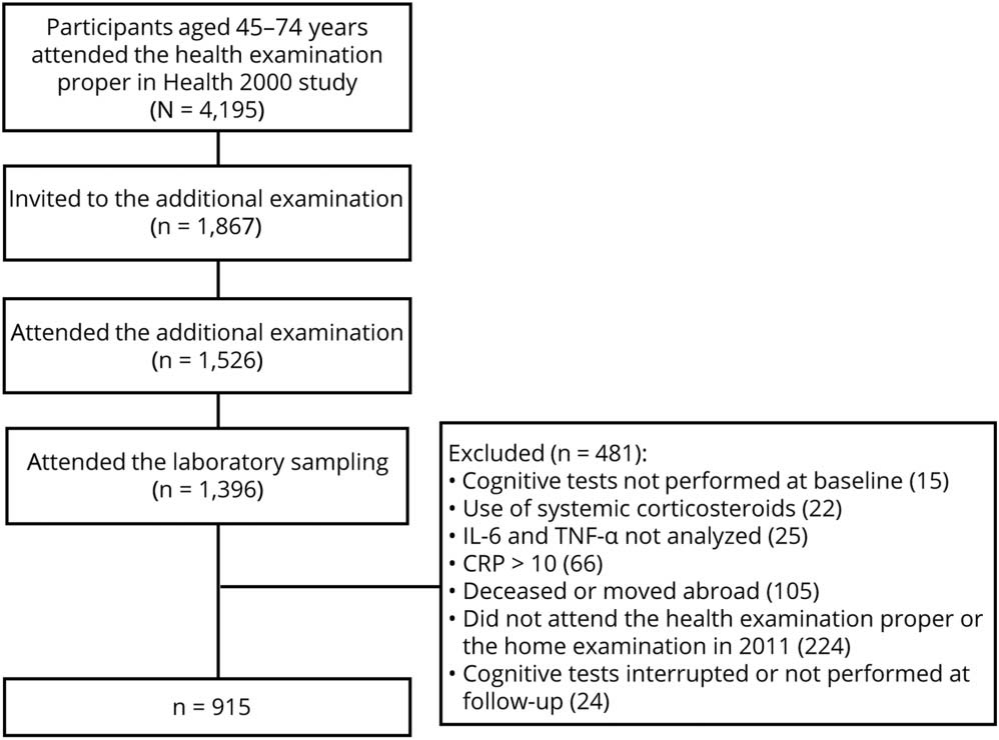
Study Selection Process and Reasons for Exclusion From the Study

Individuals who had attended all 3 investigations (the Health 2000 examination proper in 2000–2001, the supplemental investigation in 2001–2002, and the Health 2011 follow-up health examination or the home examination in 2011) were included in the present study. Participants with missing cognitive test results at baseline (n = 15) or at follow-up (n = 24) and those whose IL-6 and TNF-α -levels were not measured (n = 25) at baseline were excluded from this study. In addition, individuals with hs-CRP values > 10 mg/L (in the supplemental investigation) (n = 66) were excluded to eliminate possible confounding effects of an infectious disease. Similarly, individuals who were currently using of systemic corticosteroids (N = 22) were excluded to eliminate the potential distorting effect on inflammation markers. One hundred five individuals declined to participate in the follow-up examination or had died or were generally lost for the follow-up. In total, 915 individuals aged from 45 to 74 years were included in the analyses (Figure 1).

### Baseline Measurements

In the Health 2000 study, *APOE* genotyping was performed for those who gave their written consent for DNA sampling with the MassARRAY system (Sequenom, San Diego, CA) with a modified protocol that has previously been described.^35^ Between 2001 and 2002, participants' body mass index (BMI) was determined, and their blood pressure was measured in a sedentary position from the right upper arm 3 times with oscillometric OMRON M4 blood pressure monitor (Omron Matsusaka, Japan, OMRON Healthcare Europe B.V., Hoofddorp, The Netherlands).^36^ A mean of the 3 measurements was used in the analyses. Information about the participants' medical history, medication, education, and smoking was obtained by interviews and questionnaires. Depressive symptoms were evaluated with the Beck Depression Inventory (BDI). Fasting blood samples were taken after fasting for 10–12 hours. Serum cholesterol values were analyzed as previously reported.^33,34^ Inflammatory markers (hs-CRP, IL-6, and TNF-α) were determined using chemiluminescent immunometric assays (Immulite, Diagnostic Products Corporation, Siemens Healthcare Diagnostics, Deerfield, IL, USA). The detection limits of the assays were 0.20 mg/L for hs-CRP, 1.5 ng/L for TNF-α, and 0.5 ng/L for IL-6. Values under the detection limit were given the value detection limit divided by 2, that is, 0.1 mg/L for hs-CRP, 0.75 ng/L for TNF-α, and 0.25 ng/L for IL-6.

### Cognitive Tests at Baseline and at Follow-up

The methods used for cognitive evaluation were chosen to measure different aspects of cognition. The cognitive test battery consisted of tests for verbal fluency (VF) and learning and retaining verbal material from the Finnish version of the CERAD (Consortium to Establish a Registry for Alzheimer's Disease) test battery.^37^ Cognitive performance was measured at baseline and at follow-up.^33,34^ In the categorical VF test, the participants were asked to produce a list of as many animals as possible within 1 minute. One animal indicated 1 point. In the word-list learning (WLL) task, the participants were shown 10 words and asked to read them aloud and memorize them. Then, within 90 seconds, the participants were to recall as many words as possible (immediate recall). At baseline, this procedure was repeated twice if the participant could not recall all the words after the first round. If a participant had memorized all 10 words in the first round, the result was 30 points. Thus, the WLL score was 30 for those who recalled all the 10 words after the first round and the total of the recalled words in the 3 rounds for other participants. At follow-up, although all 10 words had been remembered after the first round, a total of 3 rounds were performed. One correct word indicated 1 point. In the word-list delayed recall (WLDR) test, the participants were asked to recall all the 10 words after a 5-minute delay.^33,34^ To evaluate the change in the cognitive test scores, the cognitive test score in 2000 was subtracted from that of 2011 so that negative change would indicate decline in the cognitive test scores during the follow-up.

### Covariates

Previously reported risk factors for cognitive decline: age, education, *APOE ε4* genotype, diabetes, hypertension, hypercholesterolemia, depressive symptoms, BMI, and smoking habits were used as covariates in the analyses. Education was determined as self-reported years of formal education. The *APOE ε4* genotype was defined as positive if the participant had 1 or 2 *APOE ε4* alleles. Hypertension was defined as systolic blood pressure ≥140 mm Hg, diastolic blood pressure ≥90 mm Hg, or current use of antihypertensive medication. Hypercholesterolemia was defined as a serum total cholesterol level ≥6.5 or current use of antilipidemic medication during the Health 2000 examinations. Diabetes was defined as the current use of insulin or oral diabetes medication, fasting plasma glucose ≥7.0 mmol/L, or 2-hour oral glucose tolerance test value ≥ 11.1. *APOE ε4*, hypertension, hypercholesterolemia, diabetes, and smoking were analyzed as dichotomic variables. Age, formal education in years, BMI, and BDI score were analyzed as continuous variables. The total number of participants with data available of each covariate is shown in Table 1. The adjusted models included all individuals with complete data for all covariates.

**Table 1:**
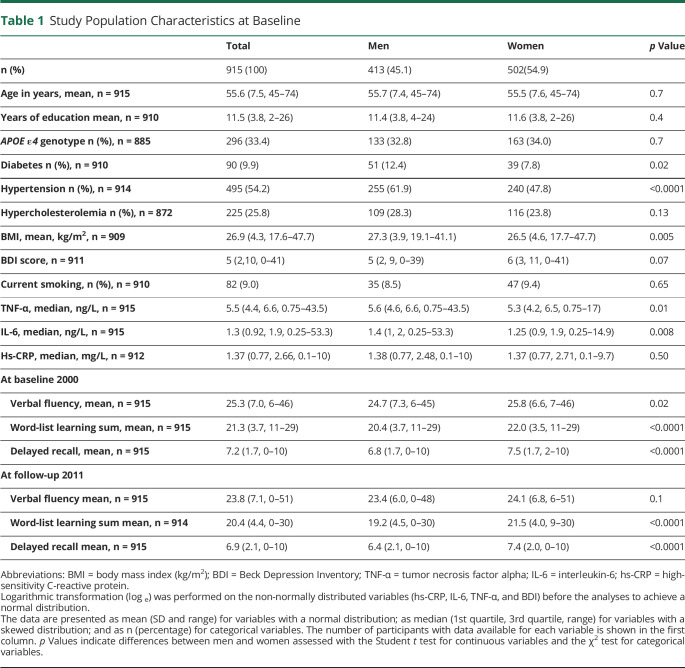
Study Population Characteristics at Baseline

### Statistical Analyses

First, the normality of all variables was inspected from the histograms. To achieve normal distribution, logarithmic transformation was performed on the non-normally distributed variables (hs-CRP, IL-6, TNF-α, and BDI). Differences between the 45-74-year-old participants of the main Health 2000 study (n = 4,195) and those included in this study (n = 915) as well as the differences between the individuals of the subpopulation who dropped out from the follow-up study (n = 353) and those who completed the follow-up (n = 915) were analyzed by the Wilcoxon rank-sum test (age), Student *t* test (other continuous variables), or χ^2^ test (sex and *APO ε4* genotype).

Because previous studies have identified differences between sexes in the associations between metabolic risk factors and cognition,^14,38^ the study population characteristics are presented for the total population and for men and women separately. Differences between men and women were analyzed with the Student *t* test for continuous variables and with the χ^2^ test for categorical variables. Pairwise correlations between inflammatory markers and covariates were calculated using Pearson correlation. Multivariable linear models were used to test the independent association between baseline inflammatory parameters and cognitive performance at follow-up. Explanatory variables were adjusted on 3 different levels. Model 1 was adjusted for age, sex, and prior education in years. Model 2 was additionally adjusted for *APOE ε4* genotype, diabetes, hypertension, and hypercholesterolemia. The fully adjusted model 3 was adjusted for all the explanatory variables above and BMI, depressive symptoms, and current smoking. The associations between inflammatory parameters and change in cognitive performance after follow-up were evaluated by adding also baseline cognitive function to the models above. Normality assumption for the analyses was verified from the studentized residuals. Age and sex interactions for each inflammatory marker on the association with cognitive performance at follow-up and with cognitive decline during the follow-up time were tested in model 1. Age group (45–65 years, n = 797, and 65–75 years, n = 118) and sex-stratified analyses were performed for the associations with significant interaction. Statistical significance was set a *p* < 0.05 for all analyses. Statistical analyses were performed with SAS JMP Pro 14 (SAS Institute, Cary; NC, USA).

### Data Availability

Anonymized data can be requested on reasonable request for a study that has been approved by a local ethics committee and that corresponds with the research areas of the Finnish Institute for Health and Welfare Biobank. The applications are to be directed to the Finnish Institute for Health and Welfare (thl.fi/en/web/thl-biobank/for-researchers).

### Standard Protocol Approvals, Registrations, and Patient Consents

The studies were approved by the Ethics Committee for Epidemiology and Public Health in the hospital district of Helsinki and Uusimaa, Finland. Each participant gave their written informed consent for participating in the studies.

## Results

### Demographics

The characteristics of the study population at baseline are presented in Table 1. The mean age of the study participants (n = 915), consisting of 413 men and 502 women, was 55.6 years; the mean BMI was 26.9 kg/m^2^ (SD 4.6). The men had higher BMI than the women 27.3 kg/m^2^ vs 26.5 kg/m^2^ (*p* = 0.005), and men had more often hypertension (61.9% vs 47, 8%, p < 0.0001) and diabetes (12.4 % vs 7.8%, p = 0.02). Two hundred ninety-six (33.4%) had 1 or 2 *APOE ε4* alleles. There was no difference in the prevalence of *APOE ε4* genotype between the men and women.

Compared with the 45-74-year-old participants of the main Health 2000 study, the participants of the present study were younger (mean age at baseline 55.6 years vs 57.3 years, *p* < 0.0001), more educated (education in years, mean 11.5 years vs 10.4 years, *p* < 0.0001), and performed better in the cognitive tests at baseline (mean VF score 25.3 vs 23.4, *p* < 0.0001; mean WLL score 21.3 vs 20.1, *p* < 0.0001; mean WLDR score 7.2 vs 6.8, *p* < 0.0001). There were no differences in sex (*p* = 0.18) or carrying *APOE ε4* genotype (*p* = 0.39). The individuals who did not attend the follow-up examinations in 2011 were older (mean age at baseline 58.9 years vs 55.6 years, *p* < 0.0001), less educated (education in years, mean 10.4 years vs 11.5 years, <0.0001), had higher levels of inflammation (median TNF-α 6.0 ng/L vs 5.5 ng/L, *p* = 0.0002; median IL-6 1.7 ng/L vs 1.3 ng/L, *p* < 0.0001; median hs-CRP 1.6 mg/L vs 1.4 mg/L, *p* < 0.0001), and performed worse in the cognitive tests at baseline (mean VF score 22.9 vs 25.3, *p* < 0.0001; mean WLL score 19.7 vs 21.3, *p* < 0.0001; mean WLDR score 6.3 vs 7.2, *p* < 0.0001) than those who finished also the follow-up examination, but there was no difference between the groups in sex (*p* = 0.43) or *APOE ε4* genotype (*p* = 0.99).

### Correlations Between Inflammatory Markers and Covariates

Elevation of each inflammatory marker was moderately correlated with the elevation of another inflammatory marker (for IL-6 and TNF-α: r = 0.31, *p* < 0.0001, for hs-CRP and TNF-α: r = 0.25, *p* < 0.0001, and for hs-CRP and IL-6: r = 0.35, *p* < 0.0001). Among the established risk factors for cognitive decline higher BMI, age, and fewer years of education, showed were associated with higher levels of all the inflammatory markers. Hs-CRP was more strongly associated with BMI than TNF-α and IL-6. Higher BDI score was associated with higher levels of IL‐6 and TNF-α (Table 2.).

**Table 2:**
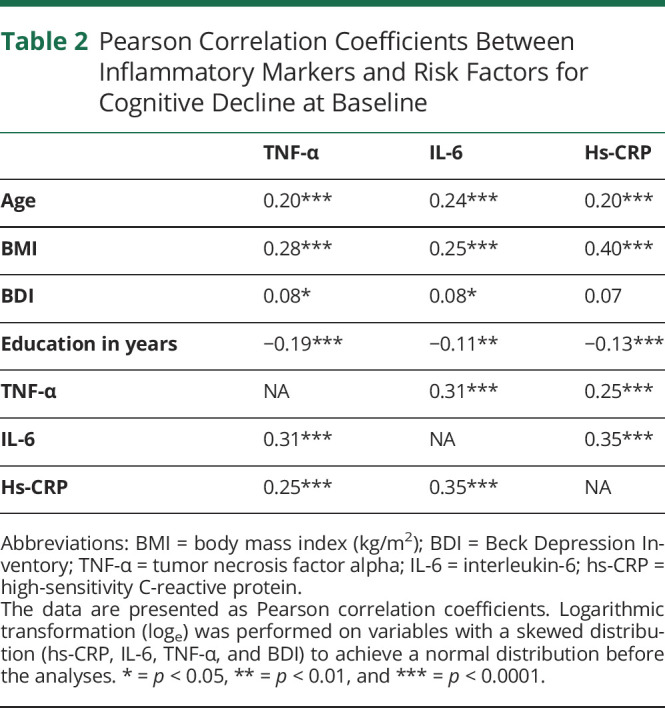
Pearson Correlation Coefficients Between Inflammatory Markers and Risk Factors for Cognitive Decline at Baseline

### Baseline Inflammatory Markers as Predictors of Follow-up Cognition

All the studied inflammatory markers predicted poorer cognitive performance at follow-up on VF, WLL, but not on WLDR, in the model adjusted only for age, sex, and education (Table 3.). After further adjustments for *APOE ε4* genotype, diabetes, hypertension, and hypercholesterolemia (model 2), these associations remained significant between TNF-α and VF (β = −2.41, *p* = 0.0007) and WLL (β = −1.03, *p* = 0.005) and between IL-6 and VF (β = −1.03, *p* = 0.004) and WLL (β = −0.60, *p* = 0.0024). In the fully adjusted model 3, a higher baseline TNF-α predicted lower VF (β = −1.78, *p* = 0.08) and WLL (β = −0.86, *p* = 0.03), and a higher baseline IL-6 predicted lower VF (β = −1.14, *p* = 0.003) and WLL (β = −0.61, *p* = 0.007). The standardized estimates for each inflammatory marker in each model are also provided in Table 3.

**Table 3:**
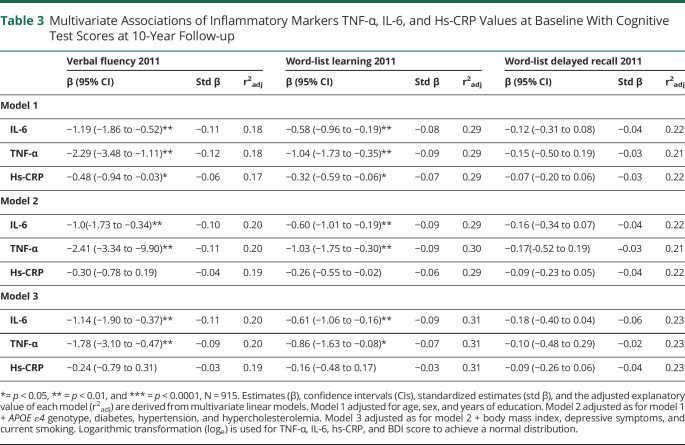
Multivariate Associations of Inflammatory Markers TNF-α, IL-6, and Hs-CRP Values at Baseline With Cognitive Test Scores at 10-Year Follow-up

### Inflammatory Markers as Predictors of Cognitive Decline From Baseline to Follow-up

In the model further adjusted for age, sex, education, and baseline cognition, TNF-α predicted a greater decline in VF (β = −1.41, *p* = 0.005) and WLL (β = −0.84, *p* = 0.007). Similarly, IL-6 predicted a decline in VF (β = −0.87, *p* = 0.002) and WLL (β = −0.52, *p* = 0.003). Hs-CRP was associated with a decline in WLL (β = −0.25, *p* = 0.04). After adjustments for the variables in model 2, higher TNF-α predicted poorer VF (β = −1.26, *p* = 0.02) and WLL (β = −0.79, *p* = 0.02). In addition, IL-6 predicted a larger decline in VF (β = −0.75, *p* = 0.01) and WLL (β = −0.52, *p* = 0.005). These associations remained significant in the fully adjusted model 3 with IL-6 and decline in VF (β = −0.81, *p* = 0.01) and WLL (β = −0.53, *p* = 0.008). In model 2 hs-CRP, and in model 3 hs-CRP and TNF-α were not associated with a decline in any of the cognitive tests (Table 4.).

**Table 4:**
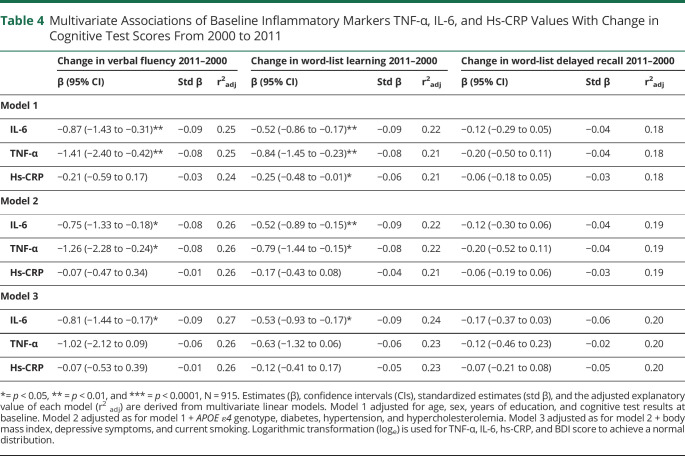
Multivariate Associations of Baseline Inflammatory Markers TNF-α, IL-6, and Hs-CRP Values With Change in Cognitive Test Scores From 2000 to 2011

### Explanatory Values of the Regression Models

Age, sex, and years of education were the driving covariates explaining variance of the cognitive test scores at follow-up (adjusted r^2^: 17% for VF, 29% for WLL, and 22% for WLDR) in a model combining these 3 covariates. Adding the inflammatory markers or the other covariates to the model improved the model only slightly (Table 3). Similar phenomena were seen when evaluating explanatory values of the a forementioned covariates and cognitive decline (Table 4). The explanatory value of a model adjusted for age, sex education, and baseline cognitive test scores was 24% for VF, 22% for WLL, and 18% for WLDR.

### Interactions

There was an interaction for age and IL-6 on the association with all 3 cognitive tests at follow-up in model 1 (VF: *p* = 0.0006; WLL: *p* = 0.003; WLDR: *p* = 0.01) and with the change in cognitive test scores (VF: *p* = 0.001; WLL: *p* = 0.004; WLDR: *p* = 0.02). No interactions were found for age and TNF-α or hs-CRP with the cognitive test scores at follow-up. In the models stratified according to age (45–65 years and over 65 years), the associations between IL-6 and VF (45–65 years: β = −1.03, *p* = 0.01; over 65 years: β = −1.26, *p* = 0.31) and IL-6 and WLL (45–65 years: β = −1.47, *p* = 0.046; over 65 years: β = −1.41, *p* = 0.11) were driven by the middle-aged population in the fully adjusted model 3. The association between IL-6 and change in VF (45–65 years: β = −0.46, *p* = 0.09; over 65 years: β = −2.33, *p* = 0.03) was stronger in the elderly population, whereas the association between IL-6 and WLL (45–65 years: β = −0.45, *p* = 0.04; over 65 years: β = −1.05, *p* = 0.17) reached statistical significance only in the younger age group. There was no association between IL-6 and WLDR in either age group.

The interaction for sex and hs-CRP was significant for WLL at follow-up (VF: *p* = 0.08; WLL: *p* = 0.004; WLDR: *p* = 0.20) and the change in WLL (VF: *p* = 0.17; WLL: *p* = 0.04; WLDR: *p* = 0.25). There were no sex interactions for IL-6 or TNF-α. However, sex-stratified analyses showed no associations between hs-CRP and WLL in model 3.

## Discussion

In this prospective cohort study, IL-6 and TNF-α independently predicted poorer performance on VF and WLL after a 10-year follow-up. Higher IL-6 in midlife also predicted a greater decline on VF and WLL over a 10-year follow-up. Hs-CRP did not have any association with the 10-year follow-up performance, nor did TNF-α or hs-CRP have any association with a decline on any of the cognitive tests in the fully adjusted model. These results suggest that TNF-α and especially IL-6 are predictors of cognitive decline. Our findings support the hypothesis that low-grade inflammation is associated with poorer cognitive performance later in life. Our findings further expand the results of previous studies indicating low-grade inflammation as a state preceding decline in cognitive functions.^8,19^ Although many studies have evaluated the predictive value of circulating inflammatory markers and cognitive decline, most of the studies have focused on elderly populations,^2^ and none of the previous prospective studies on middle-aged populations have included all 3 inflammatory markers evaluated in the present study.^8,11,12^ The present study included mostly middle-aged individuals, and the associations between low-grade inflammation and cognitive performance were somewhat stronger in the middle-aged populations in age-stratified analyses.

Few studies have reviewed the relationship between low-grade inflammation and cognition in a longitudinal prospective population-based cohort design on middle-aged individuals, and the inflammatory markers measured in these studies have varied.^9-11,13,14^ The previous studies have used only CRP,^13^ CRP combined with IL-6,^9^ elevated erythrocyte sedimentation rate (ESR),^14^ or a composite score consisting of different inflammatory markers.^10,14^ One study. followed a total of 12,336 middle-aged individuals for a 20-year period using CRP, Von Willebrand factor, fibrinogen, factor VIII, and a white blood cell count as markers for inflammation. ^10^ In line with our results, they found that a higher midlife inflammation composite score was associated with a decline in the cognitive composite score measured with multiple cognitive tests. Their domain-specific analyses revealed that a higher inflammation composite score was associated with a steeper decline in memory, but not executive function or language. Contrary to our findings, they found that elevated CRP was associated with a steeper cognitive decline over a 20-year follow-up.^10^ In the Whitehall II study (n = 5,217), an association was reported between elevated midlife IL-6 levels and cognitive decline over a 10-year follow-up measured with multiple cognitive tests.^9^ In line with our study, an association was not found between elevated CRP levels and cognitive decline.^9^ Another study followed 1719 participants for <1–8 years (mean 4.6 years, SD 0.93 years) and found that ESR predicted faster decline in verbal memory among older men.^14^ ESR was also associated with poorer performance on attention tests overall and verbal fluency among older women.^14^ The Honolulu-Asia Ageing Study (HAAS) reported a decline in cognition associated with inflammation measured with hs-CRP over 25 years of follow-up (n = 691). Cognitive performance was evaluated with the Cognitive Abilities Screening Instrument (CASI). The results remained significant only when incident dementia cases were included.^13^

Previously, the Health, Aging and Body Composition (Health ABC) study (n = 2,632) indicated an association between low-grade inflammation and cognitive decline. In the study, elderly individuals with high level inflammation and metabolic syndrome were more likely to develop cognitive impairment than those with high level of inflammation but no metabolic syndrome.^2^ In contrast, another report from the Health ABC study^11^ (n=1,323) did not find any significant association between the slope or baseline level of CRP and cognitive decline. Moreover, another study on elderly individuals (mean age 77 years) even found that increased levels of CRP were associated with a decreased risk of a drop in cognitive performance.^11,20^ One reason for inconclusive results on the relationship between elevated levels of inflammatory markers and impaired cognition in the previous studies may be that the inflammation markers and cognitive tests were measured at an older age.^2,19,20^ Given the fact that the accumulation of β-amyloid, a neuropathologic hallmark of AD, is believed to begin during midlife and that low-grade inflammation has been proposed to affect amyloid accumulation,^39^ it might be important that studies on the relationship between inflammation and cognitive impairment would include middle-aged individuals and a long follow-up period.

In the present study, higher TNF-α and IL-6 levels predicted poorer cognitive performance, whereas hs-CRP did not show a significant correlation with cognitive function. This may be due to the mechanism of synthetization and secretion of each mediator. TNF-α and IL-6 are both secreted by the adipose tissue, whereas CRP is of hepatic origin. CRP concentration increases following IL-6 secretion, and it is argued that the production of CRP is stimulated by increased IL-6 secreted from the adipose tissue of the obese.^3,40^ Therefore, the elevation of CRP levels might occur later at the inflammation cascade, possibly indicating that TNF-α and IL-6 could be more sensitive mediators of inflammation associated with the adipose tissue.

Our study had several strengths. First, we were able to evaluate the prospective associations between all 3 inflammatory markers at baseline and cognition and its decline at the follow-up. Second, the inclusion of middle-aged individuals enabled the evaluation of midlife inflammation as a predictor for cognitive decline. Third, the Health 2000 examination and its supplementary examinations included detailed information on the study participants, which made it possible to adjust the analyses for previously reported risk factors of cognitive decline. Fourth, the long follow-up time with cognitive tests both at the baseline and the follow-up allowed us to evaluate the change in cognition from midlife to older age. There are also limitations that should be taken into consideration. Inflammatory mediators were measured only once at the supplemental examinations after the original Health 2000 survey. Because the blood samples were drawn at a different time point, it is possible that there could have been fluctuation in the levels of inflammatory markers due to the effects of comorbidities or acute infections at the time of cognitive tests. However, we assume that comorbidities would not have changed notably over a 1-year time period between the cognitive tests and the measurement of inflammatory markers. In addition, the effort to exclude values that reflect acute inflammation may have led to the exclusion of hs-CRP levels reflective of high chronic inflammation.^41^ The subpopulation included in the present study was more educated, younger, and performed slightly better on the cognitive tests when compared with the original Health 2000 study population of the same age group. Similarly, those who had dropped out from the follow-up examinations had lower cognitive test scores and were less educated and older than those included in the present study. Considering these differences, however, it is likely that our results would be diluted than that we would have found false-positive associations. Another limitation to this study is that in the original Health 2000 study, no data of biomarkers or other measures of AD pathology were collected, so the relationship between AD pathology in preclinical phase and low-grade inflammation remains unknown. The final aspect that should be considered is that more sensitive measurements of cognitive performance than CERAD might have been of use in this relatively young study sample. The CERAD WLL was performed slightly differently at baseline and at follow-up. However, none of the study participants had a score of total 10 on the first round of memorizing the word list, and thus, these differences did not affect our results. In conclusion, our findings provide further evidence that a low-grade inflammation precedes and predicts the development of cognitive decline. Considering that the mean age of the study population was 55 years, these results further emphasize the role of midlife as a crucial period for predicting future cognitive decline and suggest that preventive efforts to reduce cognitive decline should be targeted at middle-aged populations.

## Glossary

AD: Alzheimer disease
IL-6: interleukin-6
hs-CRP: high-sensitivity CRP
TNF-α: tumor necrosis factor alpha
VF: verbal fluency
WLL: word-list learning
WLDR: word-list delayed recall

## References

[1] Sepehri Z, Kiani Z, Afshari M, Kohan F, Dalvand A, Ghavami S. Inflammasomes and type 2 diabetes: an updated systematic review. Immunol Lett. 2017;192:97-103. doi: 10.1016/j.imlet.2017.10.010 29079203

[2] Yaffe K, Kanaya A, Lindquist K, et al. The metabolic syndrome, inflammation, and risk of cognitive decline. J Am Med Assoc. 2004;292(18):2237-2242. doi: 10.1001/jama.292.18.2237 15536110

[3] Yudkin JS, Kumari M, Humphries SE, Mohamed-Ali V. Inflammation, obesity, stress and coronary heart disease: is interleukin-6 the link?. Atherosclerosis. 2000;148(2):209-214. doi: 10.1016/S0021-9150(99)00463-3 10657556

[4] Schindler TH, Cardenas J, Prior JO, et al. Relationship between increasing body weight, insulin resistance, inflammation, adipocytokine leptin, and coronary circulatory function. J Am Coll Cardiol. 2006;47(6):1188-1195. doi: 10.1016/j.jacc.2005.10.062 16545651

[5] Barnes DE, Yaffe K. The projected effect of risk factor reduction on Alzheimer's disease prevalence. Lancet Neurol. 2011;10(9):819-828. doi: 10.1016/S1474-4422(11)70072-2 21775213 PMC3647614

[6] Li XY, Zhang M, Xu W, et al. Midlife modifiable risk factors for dementia: a systematic review and meta-analysis of 34 prospective cohort studies. Curr Alzheimer Res. 2019;16(14):1254-1268. doi: 10.2174/1567205017666200103111253 31902364

[7] Buckley D, Fu R, Freeman M, Rogers K, Helfand M. C-reactive protein as a risk factor for coronary heart disease: a systematic review and meta-analyses for the U.S. Preventive Services Task Force. Ann Intern Med. 2009;151(7):483-95. 19805771 10.7326/0003-4819-151-7-200910060-00009

[8] Tan ZS, Beiser AS, Vasan RS, et al. Inflammatory markers and the risk of Alzheimer disease: the Framingham study. Neurology. 2007;68(22):1902-1908. doi: 10.1212/01.wnl.0000263217.36439.da 17536046

[9] Singh-Manoux A, Dugravot A, Brunner E, et al. Interleukin-6 and C-reactive protein as predictors of cognitive decline in late midlife. Neurology. 2014;83(6):486-493. doi: 10.1212/WNL.0000000000000665 24991031 PMC4141998

[10] Walker KA, Gottesman RF, Wu A, et al. Systemic inflammation during midlife and cognitive change over 20 years: the ARIC Study. Neurology. 2019;92(11):E1256–E1267. doi: 10.1212/WNL.0000000000007094 30760633 PMC6511107

[11] Metti AL, Yaffe K, Boudreau RM, et al. Trajectories of inflammatory markers and cognitive decline over 10 years. Neurobiol Aging. 2014;35(12):2785-2790. doi: 10.1016/j.neurobiolaging.2014.05.030 24997674 PMC4252870

[12] Alley DE, Crimmins EM, Karlamangla A, Hu P, Seeman TE. Inflammation and rate of cognitive change in high-functioning older adults. Journals Gerontol - Ser A Biol Sci Med Sci. 2008;63(1):50-55. doi: 10.1093/gerona/63.1.50 PMC2952346 18245760

[13] Laurin D, David Curb J, Masaki KH, White LR, Launer LJ. Midlife C-reactive protein and risk of cognitive decline: a 31-year follow-up. Neurobiol Aging. 2009;30(11):1724-1727. doi: 10.1016/j.neurobiolaging.2008.01.008 18316138 PMC7477790

[14] Beydoun MA, Dore GA, Canas JA, et al. Systemic inflammation is associated with longitudinal changes in cognitive performance among urban adults. Front Aging Neurosci. 2018;10(OCT):1-12. doi: 10.3389/fnagi.2018.00313 30356710 PMC6189312

[15] Roberts RO, Geda YE, Knopman DS, et al. Association of C-reactive protein with mild cognitive impairment. Alzheimer's Demen. 2009;5(5):398-405. doi: 10.1016/j.jalz.2009.01.025 PMC2851170 19751919

[16] Antón Alvarez X, Franco A, Fernández-Novoa L, Cacabelos R. Blood levels of histamine, IL-1β, and TNF-α in patients with mild to moderate Alzheimer disease. Mol Chem Neuropathol. 1996;29(2-3):237-252. doi: 10.1007/bf02815005 8971699

[17] Singh VK, Guthikonda P. Circulating cytokines in Alzheimer's disease. J Psychiatr Res. 1997;31(6):657-660. doi: 10.1016/S0022-3956(97)00023-X 9447570

[18] Schram MT, Euser SM, de Craen AJM, et al. Systemic markers of inflammation and cognitive decline in old age. J Am Geriatr Soc. 2007;55(5):708-716. doi: 10.1111/j.1532-5415.2007.01159.x 17493190

[19] Yaffe K, Lindquist K, Penninx, et al. Inflammatory markers and cognition in well-functioning African-American and white elders. Neurology. 2003;61(1):76-80. doi: 10.1212/01.WNL.0000073620.42047.D7 12847160

[20] Lima TAS, Adler AL, Minett T, Matthews FE, Brayne C, Marioni RE. C-reactive protein, *APOE* genotype and longitudinal cognitive change in an older population. Age and Ageing. 2014;43(2):289-292. doi: 10.1093/ageing/aft193 24305621 PMC3927773

[21] Weaver JD, Huang MH, Albert M, Harris T, Rowe JW, Seeman TE. Interleukin-6 and risk of cognitive decline: macarthur studies of successful aging. Neurology. 2002;59(3):371-378. doi: 10.1212/WNL.59.3.371 12177370

[22] Dik MG, Jonker C, Hack CE, Smit JH, Comijs HC, Eikelenboom P. Serum inflammatory proteins and cognitive decline in older persons. Neurology. 2005;64(8):1371-1377. doi: 10.1212/01.WNL.0000158281.08946.68 15851726

[23] Engelhart MJ, Geerlings MI, Meijer J, et al. Inflammatory proteins in plasma and the risk of dementia: the rotterdam study. Arch Neurol. 2004;61(5):668-672. doi: 10.1001/archneur.61.5.668 15148142

[24] Ahmed B, Sultana R, Greene MW. Adipose tissue and insulin resistance in obese. Biomed Pharmacother. 2021;137:111315. doi: 10.1016/j.biopha.2021.111315 33561645

[25] Choi J, Joseph L, Pilote L. Obesity and C-reactive protein in various populations: a systematic review and meta-analysis. Obes Rev. 2013;14(3):232-244. doi: 10.1111/obr.12003 23171381

[26] Hotamisligil GS, Shargill NS, Spiegelman BM. Adipose expression of tumor necrosis factor-α: direct role in obesity-linked insulin resistance. Science. 1993;259(5091):87-91. doi: 10.1126/science.7678183 7678183

[27] Trayhurn P, Wood IS. Adipokines: inflammation and the pleiotropic role of white adipose tissue. Br J Nutr. 2004;92(3):347-355. doi: 10.1079/bjn20041213 15469638

[28] Graßmann S, Wirsching J, Eichelmann F, Aleksandrova K. Association between peripheral adipokines and inflammation markers: a systematic review and meta-analysis. Obesity. 2017;25(10):1776-1785. doi: 10.1002/oby.21945 28834421

[29] Schäffler A, Schölmerich J, Salzberger B. Adipose tissue as an immunological organ: Toll-like receptors, C1q/TNFs and CTRPs. Trends Immunol. 2007;28(9):393-399. doi: 10.1016/j.it.2007.07.003 17681884

[30] Heneka MT, Carson MJ, Khoury Jel, et al. HHS public access neuroinflammation in Alzheimer's disease. Lancet NeurolNeuroinflammation 2018;14(4):388-405. doi: 10.1016/S1474-4422(15)70016-5 PMC5909703 25792098

[31] Toppala S, Ekblad LL, Tuisku J, et al. Association of early beta-amyloid accumulation and neuroinflammation measured with [11C]PBR28 in elderly individuals without dementia. Neurol. 2021;96(12):e1608-e1619. 10.1212/WNL.0000000000011612 PMC8032368 33514647

[32] Aromaa A, Koskinen S. Health and Functional Capacity in Finland: Baseline Results of the Health 2000 Health Examination Survey, Vol B12/2004. Publications of the National Public Health Institute; 2004:171.

[33] Lundqvist A, Mäki-Opas T, Eds. Health 2011 Survey---Methods. 2016. Finnish National Institute for Health and Welfare. Accessed February 1, 2021. http://urn.fi/URN:ISBN: 978-952-302-669-8.

[34] Heistaro S, Ed. Methodology report. Health 2000 Survey. 2008. National Public Health Institute. Accessed February 1, 2021. http://urn.fi/URN:NBN:fi-fe201204193320.

[35] Jänis MT, Siggins S, Tahvanainen E, et al. Active and low-active forms of serum phospholipid transfer protein in a normal Finnish population sample. J Lipid Res. 2004;45:2303-2309. doi: 10.1194/jlr.M400250-JLR200 15342679

[36] Menetelmäraportti KTL, Terveys 2000 -tutkimuksen toteutus, aineisto ja menetelmät. Kansanterveyslaitos; 2000. Accessed February 1, 2021. www.julkari.fi/handle/10024/78181.

[37] Lääkärilehti - kognitiiviset testit muistihäiriöiden ja alkavan dementian varhaisdiagnostiikassa: CERAD-tehtäväsarja. Accessed February 2, 2021. www.laakarilehti.fi/tieteessa/katsausartikkeli/kognitiiviset-testit-muistihairioiden-ja-alkavan-dementian-varhaisdiagnostiikassa-cerad-tehtavasarja/.

[38] Ekblad LL, Rinne JO, Puukka PJ, et al. Insulin resistance is associated with poorer verbal fluency performance in women. Diabetologia. 2015;58(11):2545-2553. doi: 10.1007/s00125-015-3715-4 26276262

[39] Villemagne VL, Burnham S, Bourgeat P, et al. Amyloid β deposition, neurodegeneration, and cognitive decline in sporadic Alzheimer's disease: a prospective cohort study. Lancet Neurol. 2013;12(4):357-367. doi: 10.1016/S1474-4422(13)70044-9 23477989

[40] Yudkin JS. Adipose tissue, insulin action and vascular disease: inflammatory signals. Int J Obes. 2003;27(S3):S25–S28. doi: 10.1038/sj.ijo.0802496 14704740

[41] mac Giollabhui N, Ellman LM, Coe CL, Byrne ML, Abramson LY, Alloy LB. To exclude or not to exclude: considerations and recommendations for C-reactive protein values higher than 10 mg/L. Brain Behav Immun. 2020;87:898-900. doi: 10.1016/j.bbi.2020.01.023 32014579 PMC7316621

